# 1. The Relationship Between Chlorhexidine Skin Concentration and Multidrug-Resistant Organism (MDRO) Colonization in ICU Patients

**DOI:** 10.1093/ofid/ofab466.001

**Published:** 2021-12-04

**Authors:** Loren G Miller, Stefan Richter, Evelyn A Flores, Sandra Ceja, Crystal Torres, Donna Phan Tran, Samantha Salcido, Gregory Tchakalian, Leah Bloomfield, Angela Gomez, Tuyen Pham, Oanh K Tong, Max Nutkiewicz, Michael Bolaris, James A McKinnell

**Affiliations:** 1 Harbor UCLA, Torrance, California; 2 Division of Infectious Diseases, the Lundquist Institute at Harbor-UCLA Medical Center, Torrance, CA, Torrance, California; 3 LA Biomed, Torrance, CA; 4 La Biomed, Los Angeles, CA; 5 MiOra, Los Angeles, CA, Los Angeles, California; 6 LA BioMed at Harbor-UCLA Medical Center, Torrance, California

## Abstract

**Background:**

Daily bathing of ICU patients with chlorhexidine gluconate (CHG) is an important method for healthcare-associated infection prevention. We set out to understand the relationship between CHG concentrations and MDRO colonization

**Methods:**

In our trauma/surgical ICU at a large urban medical center, we performed CHG concentrations 2 days/week at 4 times points relative to CHG bathing (Medline, Northfiled, IL) application: 30 min. prior, and 30 min., 6 hrs., and 12 hrs. after application. CHG testing was done at 4 body sites: lateral neck, anterior chest, arm, and inguinal fold. On the contralateral side we tested the presence of the following 4 MDROs: methicillin resistant *S. aureus* (MRSA), and 3 enteric bacteria--extended spectrum beta-lactamase (ESBL)+ gram-negative rods, vancomycin resistant enterococcus (VRE), and carbapenem resistant enterobacteriaceae (CRE).

**Results:**

We performed testing for 256 patient-days total, of which 42 were swabbed 1 time, 38 swabbed twice, 79 swabbed 3 times, and 97 swabbed 4 times (patient movement for tests, ICU transfer were limitations). Mean CHG skin concentrations were above the MICs of pathogens at all post-CHG application time points at all body sites at all times points (Figure) and decreased during the time points after bathing. In a logistic regression model controlling for patient characteristics, MRSA detection was inversely associated with CHG concentration with an 18% increase in odds of recovery for each 2-fold decrease in CHG concentration, as well as presence of a GI device and lack of ability to sit and roll. In a logistic regression model controlling for patient characteristics, resistant enteric bacteria detection was inversely associated with CHG concentration with an 11% increase in odds of recovery for each 2-fold decrease in CHG concentration, as well as mechanical ventilation, GI device, central line, and ICU duration.

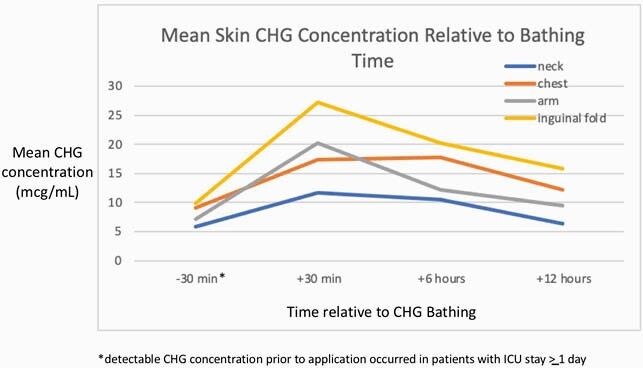

**Conclusion:**

In our large study of CHG use and its association with MDRO detection, CHG concentrations decreased during the 24 hours after application, but were typically above concentrations considered adequate to kill MDROs. CHG concentration were inversely associated with the presence of MRSA and resistant enterics, suggesting that CHG application quality is a key component of the CHG bathing process.

**Disclosures:**

**Loren G. Miller, MD, MPH**, **Medline** (Grant/Research Support, Other Financial or Material Support, Contributed product) **Stryker** (Other Financial or Material Support, Contributed product) **Xttrium** (Other Financial or Material Support, Contributed product) **James A. McKinnell, MD**, **Medline** (Grant/Research Support)

